# MRI assessment of sacral injury location and analysis of influencing factors after high-intensity focused ultrasound ablation for patients with uterine fibroids

**DOI:** 10.3389/fphys.2025.1523018

**Published:** 2025-04-16

**Authors:** Ao Zhou, Xin Feng, Furong Lv, Yunyue Tan, Yuhang Liu, Zhibo Xiao

**Affiliations:** ^1^ Department of Radiology, The First Affiliated Hospital of Chongqing Medical University, Chongqing, China; ^2^ Department of Gynaecology and Obstetrics, The First Affiliated Hospital of Chongqing Medical University, Chongqing, China

**Keywords:** ultrasound-guided high-intensity focused ultrasound, uterine fibroids, sacral injury, MRI, adverse events

## Abstract

**Purpose:**

Exploration of the location of sacral injuries following ultrasound-guided high-intensity focused ultrasound (USgHIFU) ablation for uterine fibroids and analysis of its influencing factors.

**Methods:**

A retrospective analysis was conducted on 663 patients with uterine fibroids treated by USgHIFU ablation. Patients with vertebral injuries were identified based on postoperative MRI images, with specific locations of the injuries documented. Additionally, the condition of muscle damage around the vertebral body was assessed. Patients were divided into Upper group and Lower group based on the location of vertebral injuries. Univariate and multivariate logistic regression analyses were conducted to identify the influencing factors. The χ^2^ test was used to explore the relationship between the location of vertebral injuries and postoperative clinical adverse events, as well as muscle damage.

**Results:**

Postoperative MRI examinations revealed that 42.3% (281/663) of the patients experienced vertebral injuries, which were localized to the range from L5 to S5. The injuries from L5 to S2 were classified as Upper group, accounting for 45.2% (127/281), while those from S3 to S5 were classified as Lower group, accounting for 54.8% (154/281). Multivariate analysis revealed that the distance from the ventral side of the fibroid to the abdominal wall skin, uterine position, and T2WI signal intensity were positively correlated with the location of sacral injuries (p < 0.05). Additionally, the location of sacral injuries was significantly associated with the occurrence of postoperative sacrococcygeal pain (p < 0.05). 162 patients (57.6%) with sacral injury were accompanied by piriformis and gluteus maximus muscle injuries, with piriformis injury accounting for 95.06%. The location of sacral injury was significantly correlated with piriformis injury (p < 0.05).

**Conclusion:**

Postoperative MRI images of some patients with uterine fibroids treated with USgHIFU ablation show vertebral and surrounding muscle injuries, mainly involving sacrum and piriformis. For those with a retroverted uterus, a large distance between the ventral side of the fibroid and the abdominal wall, or fibroids exhibiting high signals on T2-weighted images (T2WI), the location of postoperative sacral injuries tends to be more inferior. Additionally, these patients face an increased risk of concurrent piriformis injury and a higher likelihood of experiencing sacrococcygeal pain.

## Introduction

Uterine fibroids are the most common benign tumors of the reproductive system in women of reproductive age (Borah et al., 2013). Among them, approximately 20%–50% of individuals exhibit clinical symptoms such as increased menstrual bleeding, compressive discomfort, and pain, significantly impacting the physical and mental health of patients (Stewart et al., 2016). With advancements in medical technology, Ultrasound-guided High-Intensity Focused Ultrasound (USgHIFU) has gradually occupied an important position in the treatment of uterine fibroids due to its non-invasive nature and minimal damage to surrounding tissues. For a long time, Uterine fibroid removal surgery and uterine artery embolization are considered as alternative treatment methods for women with fertility needs to treat uterine fibroids.However, myomectomy is an invasive surgery with a relatively long postoperative recovery period, while uterine artery embolization, although less invasive, still has uncertain effects on ovarian function and pregnancy (Masciocchi et al., 2017). In contrast, USgHIFU treatment for fibroids does not require surgery, avoiding the risks of trauma and infection caused by surgery. Patients recover quickly after surgery and are usually discharged the next day. In addition, it can fully preserve the uterus and maintain the original shape of the uterine cavity to the greatest extent possible, which has a positive impact on the pregnancy outcomes of patients. Studies have shown that this method does not increase the rate of spontaneous abortion or pregnancy complications (Łoziński et al., 2019), and even the reproductive outcomes after USgHIFU treatment are superior to those after myomectomy (Anneveldt et al., 2021).Its safety and efficacy have been fully demonstrated by numerous studies (Tempany et al., 2003; Chen et al., 2018; Lee et al., 2017; Dou et al., 2024).

Nonetheless, given the complexity of lesions and human physiological structures, a minority of patients with uterine fibroids may still experience clinical adverse events after receiving USgHIFU treatment, primarily including pain and abnormal vaginal discharge, with pain being the most prominent issue (Liu et al., 2018; Chen et al., 2015). This pain encompasses multiple regions such as the sacrococcygeal area, legs, and lower abdomen, and the recovery process is relatively slow, affecting patients' quality of life. These postoperative clinical adverse events have attracted the attention of clinicians, prompting active preoperative prediction and postoperative follow-up efforts aimed at optimizing patients' treatment experiences. Magnetic Resonance Imaging (MRI), with its excellent soft tissue resolution, has become an important imaging tool for preoperative prediction and postoperative follow-up in USgHIFU treatment of uterine fibroids. Preoperatively, MRI images can be used to comprehensively assess lesion characteristics and acoustic pathway conditions, predict treatment outcomes, and formulate reasonable treatment plans. Currently, studies have incorporated radiomics to predict ablation difficulty (Wei et al., 2022). Postoperatively, the necrotic areas of targeted fibroids can be objectively evaluated to assess treatment effectiveness. In addition, MRI can also assess damage to non-target tissues. Previous studies have shown that sacral injuries can be observed on MRI images of some patients with uterine fibroids after USgHIFU, and there is a significant correlation between postoperative sacrococcygeal and leg pain and such imaging-detected injuries. Numerous studies have systematically pointed out the factors that may contribute to sacral injuries (Cun et al., 2018; Zheng et al., 2023).

Recently, Li et al. further demonstrated that there is no significant correlation between postoperative sacrococcygeal pain and the volume of sacral injury (Li et al., 2020). We understand that the vertebral bodies in different locations are adjacent to distinct soft tissue structures, and the nerve roots that traverse through the intervertebral foramina at different levels vary, each responsible for specific neural innervation areas. When vertebral injury occurs, the clinical symptoms may differ depending on the location of the injury. However, no previous reports have investigated the location of sacral injury following USgHIFU for fibroids.

Therefore, this study will analysis patients who have developed sacral injuries after undergoing USgHIFU treatment for uterine fibroids, combing MR images to record the location of sacral injury and further analyze factors that may affect the location of sacral injury. At the same time, it will evaluate the relationship between the location of sacral injury and the report of clinical adverse events after surgery.

## Materials and methods

### Patients

This study has been approved by the Ethics Committee of Chongqing Medical University in Chongqing, China (Ethics Approval Number 2021-548). As this study is retrospective, we have waived the requirement for informed consent.

We collected data from 663 patients with uterine fibroids who underwent USgHIFU ablation treatment at the First Affiliated Hospital of Chongqing Medical University from November 2017 to May 2021.

The inclusion criteria for this study are (Borah et al., 2013): confirmed patients with uterine fibroids exhibiting clinical symptoms (Stewart et al., 2016); patients who meet the criteria for HIFU treatment (Masciocchi et al., 2017); patients with complete preoperative and postoperative pelvic MRI imaging data. The exclusion criteria include (Borah et al., 2013): history of other gynecological diseases, such as endometriosis or pelvic inflammatory disease (Stewart et al., 2016); history of radiation therapy for lower abdominal malignancies (Masciocchi et al., 2017); excessively large fibroids (maximum diameter >12 cm) (Łoziński et al., 2019); pregnancy.

### Magnetic resonance imaging evaluation

Before USgHIFU treatment and within 1–2 days after treatment, all patients were examined using a 1.5T MR imaging system (Model: uMR570, Manufacturer: United Imaging Healthcare, Shanghai, China). During the examination, patients were positioned in a supine position, and the scanning area extended from the ilium to the lower edge of the pubic symphysis. The scanning parameters were set as follows: standard T1-weighted imaging (T1WI) (repetition time/echo time (TR/TE), 500/13 ms, axial plane, voxel size 1.7 × 1.3 × 5.0 mm, thickness 5 mm), T2WI (TR/TE, 5300/100 ms, axial plane, voxel size 1.0 × 1.0 × 5.0 mm, thickness 5 mm), fat-suppressed T2WI (TR/TE, 4,729/75 ms, sagittal plane, voxel size 1.0 × 1.0 × 5.0 mm, thickness 5 mm), diffusion-weighted imaging (DWI) (TR/TE, 4,800/98.8 ms, axial plane, voxel size 3.0 × 3.0 × 5.0 mm, thickness 5 mm, b-values 0 and 800 s/mm^2^), and contrast-enhanced sequences (TR/TE, 6.23/2.91 ms, axial plane, voxel size 1.7 × 1.2 × 5.0 mm, thickness 5 mm). Two experienced radiologists independently assessed the MR images of each patient, and in cases of disagreement, the final decision was made by a more senior expert within the department. The three-dimensional diameters of the non-perfused volume (NPV) and the uterine leiomyoma were measured on the enhanced images, specifically including the longitudinal diameter (D1), anteroposterior diameter (D2), and transverse diameter (D3). The volumes of the NPV and the uterine leiomyoma were calculated using the formula V = 0.5233 × D1 × D2 × D3. And several variables were derived based on the treatment parameters, including the treatment intensity (s/h, which represents the time required per hour to ablate the uterine leiomyoma using ultrasound) and the NPV ratio (NPV/leiomyoma volume). Furthermore, the following information was also recorded: the number of leiomyomas, the distances from the dorsal and ventral surfaces of the leiomyoma to the sacrum and the skin, the thickness of the abdominal wall, the position of the uterus (anteverted, mid-position, and retroverted), the location of the leiomyoma (anterior wall, posterior wall, lateral wall, and fundus), the type of leiomyoma (intramural, submucosal, and subserosal), the T2WI signal intensity (hypointense, isointense and hyperintense), and the degree of enhancement (Slight, Moderate and Significant) (Yang et al., 2021).

**FIGURE 1:**
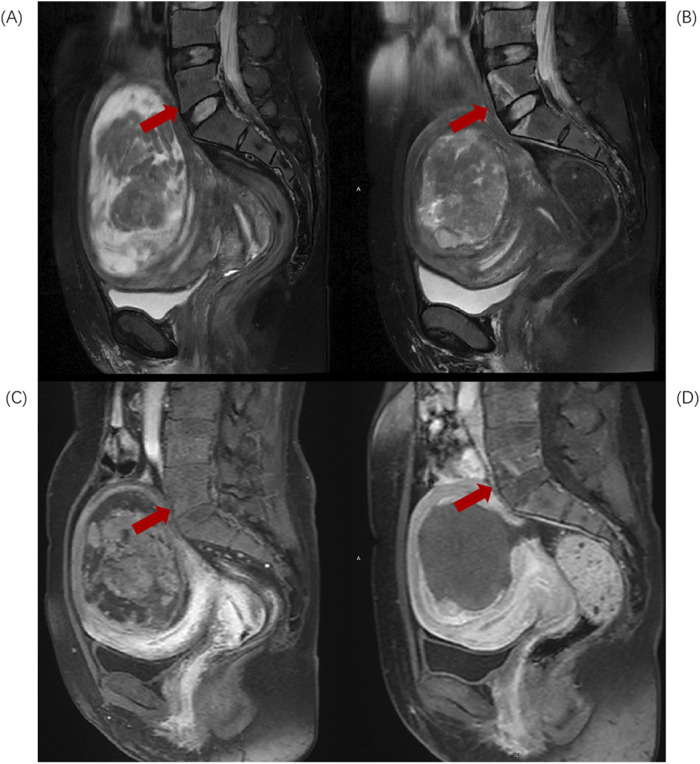
Sagittal MR images of a 43-year-old patient with uterine fibroids before and after HIFU ablation. **(A)** Pre-HIFU T2WI image shows normal signal intensity in L5 and S1 (arrows); **(B)** Post-HIFU T2WI shows patchy mixed high and low signal areas in L5 and S1 (arrows); **(C)** Pre-HIFU enhanced imaging shows normal enhancement in L5 and S1 (arrows); **(D)** Post-HIFU enhanced image shows decreased perfusion in the mixed high and low signal area of L5 and L1 on T2WI (arrow).

**FIGURE 2:**
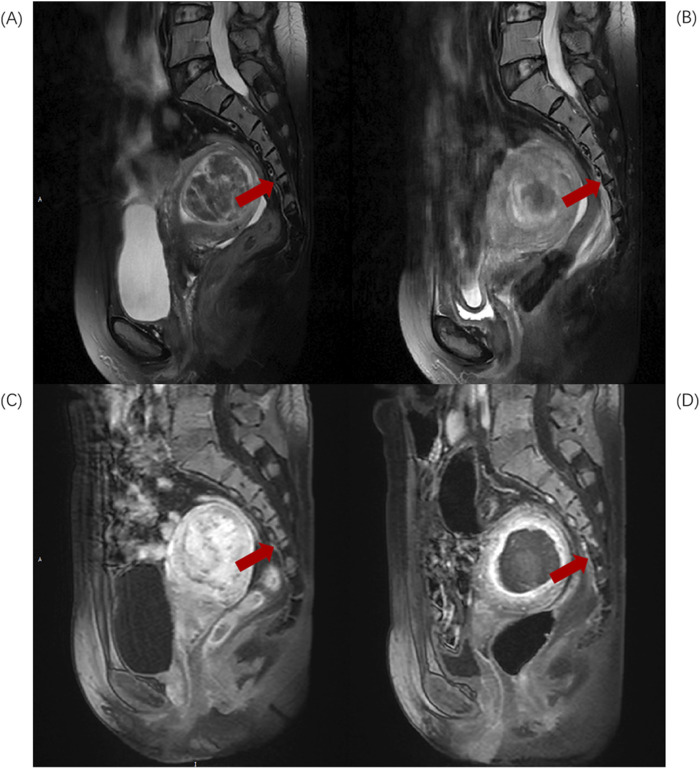
Sagittal MR images of a 46-year-old patient with uterine fibroids before and after HIFU ablation. **(A)** Pre-HIFU T2WI image shows normal signal intensity in S4 and S5 (arrows); **(B)** Post-HIFU T2WI shows patchy mixed low signal areas in S4 and S5 (arrows); **(C)** Pre-HIFU enhanced imaging shows normal enhancement in S4 and S5 (arrows); **(D)** Post-HIFU enhanced image shows decreased perfusion in the mixed low signal area of S4 and S5 on T2WI (arrow).

Based on the anatomical characteristics of the lumbosacral region and the needs of injury assessment, L5-S2 is considered the upper region of the sacrum, while S3-S5 constitute the lower region of the sacrum. When the injury involves both the upper and lower regions, the “location of the largest volume of injury” is used as the criterion for defining the position of the sacral injury. Divide L5-S2 injuries into Upper group (Figure 1) and S3-S5 injuries into Lower group (Figure 2).

### USgHIFU ablation

All treatments were performed using the JC-type focused ultrasound tumor treatment system produced by Chongqing Haifu Medical Technology Co., Ltd. in China for tumor ablation. The ultrasonic transducer equipped in this system operates at a frequency of 0.8 MHz, with a focal length of 15 cm and a diameter of 20 cm. The ablation process was conducted under the real-time guidance of the Mylab 70 ultrasound imaging system manufactured by Esaote, an Italian company, which ensured precise target positioning and monitoring of treatment effects. During the surgery, the ablation power was maintained constantly at 300–400 W, and the treatment started from the posterior part of the uterine leiomyoma, with the starting point at least 10 mm away from the dorsal edge of the tumor. The treatment parameters, including ultrasonic power and duration, were dynamically adjusted based on the patient’s immediate feedback and changes in grayscale of the target area’s ultrasonographic images. When the monitoring ultrasound indicated the disappearance of blood flow perfusion signs within the target tissue or significant changes in grayscale of the color Doppler ultrasound images, it was considered that the treatment was completed and could be terminated. At this point, various treatment parameters and adverse reaction indicators were recorded and calculated, including the sonication power, sonication time, treatment time, treatment intensity (s/hour), therapeutic dose (TD), non perfused volume (NPV), non perfused volume ratio (NPVR),and energy efficiency factor (EEF, J/mm^3^, energy required per unit volume of fibroid ablation).

During the treatment, the patient lay in a prone position on the treatment table, with their abdominal wall in contact with the ultrasonic transducer through circulating degassed water (maintained at a temperature below 15°C). The patient was kept in a conscious and sedated state after intravenous injection of fentanyl citrate and midazolam hydrochloride. Throughout the entire procedure, the patient needed to remain awake to communicate effectively with the physician and report any discomfort promptly. All feedback was meticulously recorded. The severity of post-operative adverse events was recorded according to the classification system of the Society of Interventional Radiology (SIR). The SIR classification criteria are as follows: Category A involves no treatment and no consequences; Category B requires nominal treatment or no substantive consequences, with only overnight observation needed; Category C necessitates treatment and minor hospitalization (<48 h); Category D requires major treatment, including unplanned escalation of care level or prolonged hospitalization (>48 h); Category E refers to permanent adverse sequelae; and Category F indicates death (Cardella et al., 2009)^.^

### Statistical analysis

Data analysis was performed using SPSS 26.0 statistical software (IBM, United States). Count data were expressed as frequencies, normally distributed data as means ± standard deviations, and skewed distributed data as medians with interquartile ranges. The location of sacral injury served as the dependent variable, while fibroid characteristics and treatment parameters were considered independent variables. Univariate analysis was conducted using independent sample t-tests, Mann Whitney tests, and χ^2^ tests. Variables with p < 0.05 in the univariate analysis underwent further collinearity testing to ensure absence of serious collinearity issues. Subsequently, these variables were included in an unconditional binary logistic regression model for multivariate analysis. The relationships between postoperative clinical adverse events and the location of sacral injury, as well as between the location of sacral injury and surrounding muscular soft tissue injury, was analyzed by χ^2^ tests. A P-value <0.05 was considered statistically significant.

## Results

### Baseline characteristics and fibroid features of the patients

A total of 663 patients with uterine fibroids who underwent USgHIFU ablation treatment were enrolled, with a median age of 42 years (range: 35–45 years). The maximum diameter and volume of the fibroids were 6.23 cm (range: 5.12 cm–7.42 cm) and 90.48 cm^3^ (range: 52.59 cm^3^–167.04 cm^3^), respectively. Compared with preoperative MR images, postoperative MRI examination found that 281 patients (42.3%, 281/663) had vertebral body injuries, which were limited to the range of L5 to S5. On T2WI, they showed patchy high or low signal, and on CE-MRI, they showed weak or no enhancement. Non perfusion volume ratio (NPVR) is a key indicator for evaluating the effectiveness of USgHIFU ablation of uterine fibroids, which directly reflects the degree of damage to the lesion tissue caused by treatment. The higher the NPVR value, the larger the non perfusion area within the fibroid tissue, that is, the wider the range of coagulative necrosis, and the better the treatment effect. The statistical analysis results showed that the NPVR of patients with vertebral injuries was 76.2% (63.9%–88.1%), while the NPVR of patients without vertebral injuries was 76.6% (63.5%–86.3%). There was no significant statistical difference between the two groups (P > 0.05, Table 1).

**TABLE 1:** Comparison of USgHIFU ablation effects between vertebral body injury group and non injury group.

Characteristics	Vertebral body injury group (n = 281)	Non injury group (n = 382)	P value
NPV ratio (%)	0.76 (0.64–0.88)	0.77 (0.63–0.86)	0.593
NPV (cm^3^)	65.80 (40.05–125.47)	58.74 (34.47–112.24)	0.053

Among the damaged vertebrae, S2 and S3 vertebrae accounted for 66% and 76% of the patients, respectively, while the other affected vertebrae were L5 (9%), S1 (37%), S4 (56%), and S5 (23%). According to the location of the damaged vertebral body, L5-S2 injury was divided into Upper group, accounting for 45.2% (127/281); S3-S5 injuries were divided into Lower group, accounting for 54.8% (154/281).

In the lower group, the median distance from the ventral surface of the fibroid to the skin was 51.08 mm (interquartile range: 36.93 mm–68.86 mm), compared to 39.58 mm (interquartile range: 31.03 mm–55.46 mm) in the upper group. Notably, the proportion of patients with uterine retroversion and high signal intensity on T2-weighted images (T2WI) was significantly higher in the lower group compared to the upper group. Statistically significant differences were observed between the two groups in terms of the distance from the ventral side of the fibroid to the abdominal wall, the maximum diameter and volume of the fibroid, uterine position, and T2WI signal intensity (p < 0.05, Table 2). No significant differences were observed in other variables between the two groups (p > 0.05).

**TABLE 2:** Univariate analysis to evaluate the relationship between the location of sacral injury and fibroid features.

Characteristics	Lower group (n = 154)	Upper group (n = 127)	p value
Age (years)	42.5 (37–46)	43 (38–46)	0.187^a^
Thickness of abdominal wall (mm)	25.98 (20.92–30.57)	25.05 (19.79–29.34)	0.219^a^
Fibroid ventral side to the skin (mm)	51.08 (36.93–68.86)	39.58 (31.03–55.46)	<0.01^a^
Fibroid dorsal side to the sacrum (mm)	20.54 (13.29–33.52)	21.02 (13.31–36.39)	0.542^a^
Maximal diameter (cm)	59.96 (50.91–73.25)	66.1 (54.81–78.85)	0.028^a^
Volume of fibroid (cm^3^)	85.87 (51.92–166.17)	112.43 (66.86–195.25)	0.019^a^
Number of fibroids	2 (1–3)	2 (1–3)	0.123^a^
Position of the uterus, n (%)			<0.01^b^
anteverted	83 (53,9)	102 (80.3)	
mid-position	19 (17.5)	13 (10.2)	
retroverted	52 (33.8)	12 (9.4)	
Location of fibroids, n (%)			0.185^b^
anterior wall	40 (26.0)	41 (32.3)	
posterior wall	69 (44.8)	41 (32.3)	
lateral wall	35 (22.7)	33 (26.0)	
fundus	10 (6.5)	12 (9.4)	
Type of fibroid, n (%)			0.873^b^
intramural	126 (81.8)	101 (79.5)	
submucosal	9 (5.8)	9 (7.1)	
subserosal	19 (12.3)	17 (13.4)	
T2WI, n (%)			0.014^b^
hypointense	24 (15.6)	20 (15.7)	
isointense	63 (40.9)	72 (56.7)	
hyperintense	67 (43.5)	35 (27.6)	
Degree of enhancement, n (%)			0.155^b^
Slight	54 (35.1)	40 (31.5)	
Moderate	56 (36.4)	60 (47.2)	
Significant	44 (28.6)	27 (21.3)	

T2WI, T2-weighted imaging; n, number of patients.

^a^ From the Mann Whitney U-test.

^b^ From the χ2 test.

### Comparison of USgHIFU treatment parameters between the two groups

The median EEF for the upper and lower sacral injury groups were 5.25 J/mm^3^ (range 3.97–8.25) and 4.67 J/mm^3^ (range 3.26–6.19), respectively, with median TD for ablation treatment being 520 KJ (range 384.00–790.80) and 440 KJ (range 298.40–641.13), respectively. As shown in Table 3, there were statistically significant differences between the upper and lower sacral injury groups in terms of treatment time, sonication time, TD, EEF, and NPV (p < 0.05). However, there were no statistically significant differences between the two groups in terms of sonication power, treatment intensity, and NPVR (p > 0.05).

**TABLE 3:** Univariate analysis to evaluate the relationship between the location of sacral injury and ultrasound ablation parameters.

Variables	Lower group (n = 154)	Upper group (n = 127)	P value
Sonication power (W)	400 (400–400)	400 (400–400)	0.444^a^
Sonication time (s)	1,103 (750–1744)	1,400 (1,038–2000)	<0.01^a^
Treatment time (min)	113 (80–148)	131 (102–170)	<0.01^a^
Treatment intensity (s/h)	626.05 ± 164.47	656.20 ± 158.71	0.110^b^
TD (KJ)	440 (298.40–641.13)	520 (384.00–790.80)	<0.01^a^
NPV (cm^3^)	60.58 (33.23–111.10)	76.28 (49.06–140.86)	0.024^a^
EEF (J/mm3)	4.67 (3.26–6.19)	5.25 (3.97–8.25)	<0.01 ^a^
NPVR (%)	0.75 (0.64–0.88)	0.77 (0.65–0.89)	0.742a

TD, therapeutic dose; NPV, non-perfused volume; NPV, ratio, the ratio (%) of NPV-to-fibroid volume; EEF, energy efficiency factor.

In each category, numbers in the parentheses indicate the range.

^a^ From the Mann Whitney U-test.

^b^ From the independent t-test.

### Multivariate analysis of factors affecting the location of sacral injury

In the univariate analysis, we observed significant differences between the upper and lower sacral injury groups in terms of distance from the ventral side of the fibroid to the skin, maximum diameter of the fibroid, fibroid volume, uterine position, T2WI signal intensity, treatment time, sonication time, TD, EEF, and NPV. Further collinearity diagnosis showed sonication time (VIF = 22.430), fibroid volume (VIF = 21.320), TD (VIF = 19.087), and NPV (VIF = 11.998). Based on Variance Inflation Factor (VIF), Gradually remove sonication time and fibroid volume, and rerun the regression model to calculate the VIF values of the remaining variables. At this point, the VIF values of all independent variables are within an acceptable range, thus avoiding the influence of multicollinearity. Subsequently, the distance from the ventral side of the fibroid to the skin, maximum diameter of the fibroid, uterine position, T2WI signal intensity, treatment time, TD, NPV, and EEF selected for binary logistic regression (Enter) in the multivariate analysis. The final results indicate that the distance from the ventral side of the fibroid to the skin, uterine position, and T2WI signal intensity are significantly correlated with the location of sacral injuries. Compared to patients with anteverted uteri, those with retroverted uteri are at a higher risk of sustaining injuries to the lower part of the sacrum following USgHIFU procedures for fibroids (OR = 4.362, p < 0.001). Additionally, fibroids with high T2WI signals and an increased distance from the ventral side of the fibroid to the abdominal wall skin are positively correlated with injuries to the lower sacrum (p < 0.05, Table 4).

**TABLE 4:** Multivariable binary logistic regression analysis to evaluate the correlation of the location of sacral injury with the significant factors of univariate analysis.

Variables	B	SE	Wald	p	Ors [Exp(B)]	95%CI		Collinearity statistics	
						Lower	Upper	Tolerance	VIF
Fibroid ventral side to the skin (mm)	0.019	0.008	6.044	0.014	1.019	1.004	1.034	0.745	1.342
Maximal diameter (cm)	−0.002	0.014	0.013	0.908	0.998	0.972	1.026	0.223	4.492
Treatment time (min)	−0.005	0.005	1.240	0.266	0.995	0.985	1.004	0.298	3.361
NPV (cm^3^)	0.000	0.003	0.008	0.927	1.000	0.995	1.005	0.244	4.091
EEF (J/mm3)	−0.040	0.043	0.879	0.348	0.960	0.883	1.045	0.486	2.057
TD (KJ)	0.000	0.001	0.129	0.720	1.000	0.998	1.002	0.233	4.284
Position of the uterus, n (%)			15.146	0.001				0.893	1.120
anteverted^a^									
mid-position	0.553	0.428	1.669	0.196	1.738	0.751	4.019		
retroverted	1.473	0.384	14.741	0.000	4.362	2.057	9.252		
T2WI,n (%)			6.370	0.041				0.788	1.269
hypointense^a^									
isointense	0.328	0.433	0.575	0.448	1.389	0.594	3.246		
hyperintense	0.922	0.425	4.699	0.030	2.514	1.092	5.785		

^a^ Show comparison items.

### Clinical adverse events

Among the 281 patients with sacral injuries in this study, 51.2% (144/281) reported postoperative clinical adverse reactions, with an incidence of clinically adverse events requiring intervention of 23.4% (66/281). The postoperative clinical adverse events were graded and assessed according to the SIR (Society of Interventional Radiology) criteria. The results showed that only Grade A and Grade B clinical adverse events were observed in this study, with incidence rates of 31.6% (89/281) and 23.4% (66/281), respectively. Among the Grade A adverse events, abnormal vaginal secretion was the most common, with an incidence rate of 27.5% (35/127) in the upper sacral injury group and 18.1% (28/154) in the lower group. These Grade A adverse events resolved spontaneously within a short period of time without any treatment. As for Grade B adverse events, the incidence rate of sacrococcygeal pain was 2.3% (3/127) in the upper group, while it was higher in the lower group, at 7.1% (11/154). Statistical analysis revealed a significant difference in the incidence rate of sacrococcygeal pain between the upper and lower sacral injury groups (p < 0.05, Table 5), indicating that the lower group was more prone to this symptom. For other types of adverse events, no significant correlation was observed in their incidence rates between the two groups (p > 0.05, Table 5).

**TABLE 5:** Postoperative adverse effects.

SIR classification	Adverse event	Lower group (n = 154)	Upper group (n = 127)	p Value
Class A	Vaginal discharge	28 (18.1%)	35 (27.5%)	0.109
	Lower abdominal pain	3 (1.9%)	6 (4.7%)	0.254
	Sacrococcygeal pain/	9 (5.8%)	4 (3.1%)	0.106
	Nausea and vomiting	1 (0.6%)	1 (0.7%)	0.747
	Odynuria	1 (0.6)	0 (0%)	0.494
	Erythema on skin	0 (0%)	1 (0.7%)	0.506
Class B	Vaginal discharge	13 (8.4%)	10 (7.8%)	0.420
	Sacrococcygeal pain	11 (7.1%)	3 (2.3%)	0.025
	Lower limb numbness/pain	4 (2.5%)	1 (0.7%)	0.212
	Erythema on skin	1 (0.6%)	2 (1.5%)	0.458
	Lower abdominal pain	5 (3.2%)	7 (5.5%)	0.294
	Odynuria	1 (0.6%)	1 (0.7%)	0.727
	Nausea and vomiting	3 (1.9%)	4 (3.1%)	0.437
Class C	—	0	0	—
Class D-F	—	0	0	—

SIR, society of interventional radiology.

### Injury of muscles around the sacrum

**FIGURE 3:**
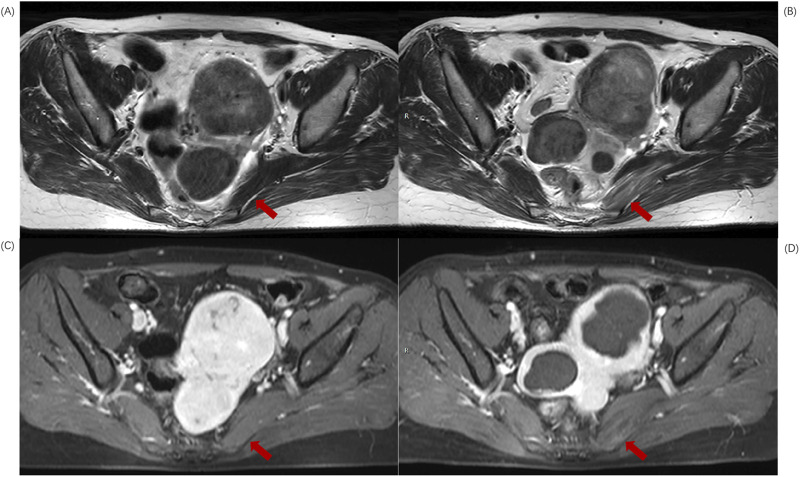
MR transverse sectional image of a 39-year-old patient with uterine fibroids before and after HIFU ablation. **(A)** Pre-HIFU T2WI image shows normal signal intensity in left piriformis muscle (arrows); **(B)** Post-HIFU T2WI shows mixed high signal areas in left piriformis muscle (arrows); **(C)** Pre-HIFU enhanced imaging shows normal enhancement in left piriformis muscle (arrows); **(D)** Post-HIFU enhanced image shows decreased perfusion in the mixed high signal area of left piriformis muscle on T2WI (arrow).

In this study, 57.2% (162/281) of patients with sacral injuries also had concurrent surrounding muscle damage (Figure 3).The piriformis muscle was the most commonly injured muscle, accounting for 95.06% (154/162) of cases, while gluteus maximus muscle damage accounted for 4.94% (8/162), and other muscle damages accounted for 0%. The incidence of piriformis muscle damage was 31.5% (40/127) in the upper sacral injury group, while it increased to 74.1% (114/154) in the lower group. As shown in Table 6, there was a significant correlation between the location of sacral injury and piriformis muscle damage (p < 0.05), while there was no statistically significant difference between the location of sacral injury and gluteus maximus muscle damage (p > 0.05).

**TABLE 6:** Univariate analysis of the correlation between sacral injury location and surrounding muscle injury.

Characteristics	Lower group (n = 154)	Upper group (n = 127)	P value
Pear shaped muscle injury, n (%)			<0.01
without	40 (25.9%)	87 (68.5%)	
with	114 (74.1%)	40 (31.5%)	
Gluteus maximus muscle injury, n (%)			0.059
without	147 (95.4%)	126 (99.2%)	
with	7 (4.6%)	1 (0.8%)	

## Discussion

Through extensive clinical practice and research validation, USgHIFU treatment for uterine fibroids has been proven to be a minimally invasive procedure with high safety. However, due to the reflection and refraction of ultrasound beams when passing through different interfaces with significant differences in acoustic impedance (Laugier and Haïat, 2011), this may lead to energy accumulation outside the target area, causing damage to surrounding local tissues, which can be effectively detected by MRI. Cun et al. reported that among 346 patients with single fibroid, 135 (39.0%) showed changes in sacral MRI signal intensity after USgHIFU^[12]^. The research results of Li et al. showed that among 267 patients with uterine fibroids treated with USgHIFU, 87 cases (32.6%) showed sacral signal changes after surgery ^[14]^.

In this study, we conducted follow-up MR examinations on 663 patients with uterine fibroids who underwent USgHIFU treatment. The results showed that 281 patients had observed sacral injuries, with an injury incidence rate as 42.3% (281/663). The median NPVR of patients with sacral injury was 76.2% (63.9%–88.1%), while the NPVR of patients without sacral injury was 76.6% (63.5%–86.3%), both achieving good therapeutic effects (Zheng et al., 2023). There was no significant statistical difference between the two groups, and the presence of imaging damage to the vertebral body after surgery had no significant impact on the treatment effect. Further analysis revealed that 23.4% (66/281) of patients with sacral injuries experienced clinical adverse events requiring intervention, indicating that only a small portion of patients with imaging detected injuries would experience clinical complications requiring intervention. This result is consistent with previous studies.In this study, we found that postoperative injuries can occur in vertebral bodies such as L5-S5, the S2 and S3 vertebrae were the most frequently affected by thermal damage. Among them, 154 patients (54.8%) mainly presented with injuries in the lower S3-S5 region, while another 127 patients (45.2%) had injuries in the upper L5-S2 region.

Further research findings revealed significant differences between the up and low vertebral injury groups in terms of the distance from the ventral side of the fibroid to the skin, the maximum diameter of the fibroid, fibroid volume, uterine position, T2WI signal intensity, treatment time, sonication time, thermal dose (TD), energy efficiency factor (EEF), and non-perfused volume (NPV). However, only uterine position, the distance from the ventral side of the fibroid to the skin, and T2WI signal intensity were found to influence the location of vertebral injuries. Specifically, a retroverted uterus, an increased distance from the ventral side of the fibroid to the abdominal wall skin, and a fibroid with high T2WI signal intensity were more likely to result in lower vertebral injuries. Myomas that exhibit high signal intensity on T2WI are characterized by their high water content and relatively low collagen content. This composition results in poor ability to absorb ultrasonic energy and convert it into thermal energy, thus making treatment more challenging and requiring higher irradiation energy to achieve therapeutic effects (Zhao et al., 2015; Gong et al., 2021). As a result, the required energy input increases. When the uterus tilts backwards and/or the distance from the abdominal side of the fibroid to the abdominal wall skin increases, it means that the target fibroid is located at a deeper position. It is necessary to increase the incidence angle of ultrasound during treatment to ensure that the beam can be accurately focused on the target fibroid during ablation. Due to the fact that ultrasound undergoes refraction when passing through different tissue interfaces, and the refraction angle is closely related to the incident angle. Therefore, an increase in the incident angle necessarily leads to an increase in the refraction angle, causing the propagation axis of the posterior ultrasound wave to be primarily directed towards the lower sacral bone. Given that the focal energy always extends along the propagation axis of the ultrasound wave, the lower sacral bone will receive more thermal energy. Moreover, as the primary load-bearing structure of the sacrococcygeal region, the high-positioned vertebral bodies are typically wide. In contrast, the low-positioned sacral vertebrae are anatomically smaller in volume, implying a smaller surface area for heat dissipation. Additionally, their vascular distribution is relatively sparse, and blood flow velocity is slower (Gailloud, 2019). The flowing blood has a heat dissipation effect, which can effectively dissipate the heat in the target area (Marinova et al., 2021). Therefore, the energy of the light beam deposited in the low-positioned vertebral body is more difficult to effectively disperse.Coupled with the dual factors of increased energy input and heat absorption, the low-positioned vertebral bodies are thus more susceptible to damage.

Safety remains a critical concern in the treatment of uterine fibroids with USgHIFU. The incidence of clinical adverse events observed in this study is comparable to that reported in previous studies, among which postoperative sacrococcygeal pain significantly impacts patients' daily lives. The results of this study indicate that there is a significant correlation between postoperative sacrococcygeal pain and the location of vertebral injury. Patients are more likely to experience postoperative sacrococcygeal pain when the vertebral injury is located at a lower position. The sacral plexus nerves are located in the sacrococcygeal region and serve as a crucial component of the spinal nerves, responsible for innervating the sensations and movements of the pelvic region, buttocks, perineum, and lower limbs. Once damaged or stimulated, they may trigger a series of symptoms such as pain, numbness, and paresthesia. Therefore, postoperative sacrococcygeal pain may be associated with intraoperative ultrasonic energy stimulation of the sacral nerves. However, currently, there is no direct medical research demonstrating absolute differences in the abundance or sensitivity of nerve endings surrounding the up and low sacrum. Therefore, the increased likelihood of sacrococcygeal pain following low sacral injury cannot be directly attributed to sacral nerve stimulation. Thermal injury to the vertebral body not only directly affects the bone tissue but may also induce swelling and degeneration of adjacent soft tissues. The soft tissues adjacent to the up and low vertebral bodies exhibit significant differences in anatomical location, physiological function, and potentially associated diseases. We speculate that these differences are more likely to be the reason why patients with low sacral injuries are prone to experiencing sacrococcygeal pain.

Our results of this study further support this speculation. We found that among 281 patients with sacral injuries, 57.6% (162/281) developed concurrent injuries to the surrounding muscular soft tissues, with piriformis muscle injury being the most common. Statistical analysis revealed a significant correlation between the location of sacral injury and piriformis muscle injury: injuries to the lower vertebrae were more likely to be accompanied by piriformis muscle damage. This may be related to the anatomical characteristics of the piriformis muscle, whose origin is primarily located on the anterior surface of the lower sacrum and is tightly attached to the lower sacrum. Consequently, when the lower sacrum sustains thermal injury, heat energy can be directly transmitted to the piriformis muscle along these attachment points, thereby increasing the risk of its damage.The piriformis muscle exits the pelvis through the greater sciatic foramen and enters the gluteal region, with important structures such as the sciatic nerve, other sacral plexus nerves, and gluteal blood vessels passing through its exit. During ablation procedures, excessive thermal deposition in the piriformis muscle region can lead to edema and degeneration. When the edema intensifies to a certain extent, it can cause narrowing of the piriformis muscle exit, subsequently leading to traction and compression of the nerves and blood vessels passing through it, ultimately inducing sacrococcygeal pain. This discovery provides a new perspective for understanding the mechanisms underlying postoperative sacrococcygeal pain.

With appropriate treatment, the clinical complications typically resolve within a week and have no substantial long-term impact on patients. For patients injuries detected by imaging but no clinical symptoms, a clinical follow-up strategy is recommended. Through regular clinical follow-up observations, changes in the patient’s condition can be promptly understood. If any clinical symptoms appear during the follow-up process, further MRI imaging follow-up should be conducted to more accurately assess the injury and administer medication appropriately based on the specific situation, ensuring timely detection and proper management of any potential clinical symptoms, and maximizing the control of the occurrence and development of clinical adverse events.

For high-risk fibroid patients with retroverted uterus, large distance between fibroids and abdominal skin, and high signal on T2WI, while ensuring the ablation effect, doctors can place the ultrasound probe in a relatively lower position in the abdomen and adjust its angle appropriately to tilt upwards, that is, away from the lower sacrum direction. This adjustment aims to change the propagation path of ultrasound in the pelvic cavity, so that the posterior ultrasound can avoid the lower sacral area as much as possible, thereby reducing the risk of injury. During the treatment process, doctors need to utilize real-time monitoring methods such as ultrasound imaging and temperature monitoring to observe the treatment progress and flexibly adjust treatment parameters, including sonication power and treatment time, based on actual needs, in order to reduce energy deposition. When necessary, a staged treatment strategy can be adopted, with initial treatment focusing on the anterior region of the fibroid, which is relatively distant from bony structures. After several months, as the fibroid necroses and shrinks, increasing the safe distance between the fibroid and the sacrum, subsequent treatment can be administered to ensure safety. Simultaneously, the clinical team should proactively inform patients that, due to the specific characteristics of their fibroids, they may be at a higher risk of experiencing pain postoperatively, in order to help them prepare psychologically. By discussing these mitigation strategies, we aim to provide clinicians with more specific and feasible operational guidance and suggestions. Although the actual improvement effects of these strategies remain to be verified by future clinical practice, actively exploring solutions has always been an important topic worthy of attention in clinical research.

In recent years, various advanced CNN (Convolutional Neural Network) algorithm architectures have emerged in the field of deep learning, demonstrating tremendous application potential in the assessment of complex anatomical structures such as vertebrae and muscles (Iqbal et al., 2020a; Iqbal et al., 2020b). In the future, we should actively explore and integrate these cutting-edge technologies, striving to reduce the influence of subjective factors in the evaluation of non-target tissue damage after USgHIFU surgery, and achieve accurate prediction or quantification of the severity of damage. At the same time, we should closely correlate imaging results with actual clinical outcomes, and develop a real-time, efficient decision support system for the formulation of USgHIFU treatment plans and postoperative care.

The present study is a single-center, retrospective study that employs a manual radiological assessment method for injury evaluation, which has issues of accuracy and consistency. Additionally, the study did not explicitly explore individual differences in pain tolerance, which may have an impact on the reporting and rating of patient treatment responses. Therefore, the results of this study have certain limitations. In the future, it is necessary to conduct high-quality multicenter studies with the assistance of deep learning models to further validate and refine the findings.

In summary, postoperative MRI images of some patients with uterine fibroids treated with USgHIFU ablation show vertebral and surrounding muscle injuries, mainly involving sacrum and piriformis. The lower the position of the sacral injury, the more likely it is to be accompanied by piriformis muscle damage, and postoperative sacrococcygeal pain symptoms are more likely to occur. Therefore, the location of sacral injury is an easily observable and crucial observation item in the MR evaluation after USgHIFU ablation for uterine fibroids. The factors influencing the location of postoperative sacral injury include the distance from the ventral side of the fibroid to the abdominal wall, the position of the uterus, and the signal intensity on T2-weighted imaging (T2WI). Taking these factors into full consideration can help optimize the preoperative assessment process for USgHIFU treatment of uterine fibroids and more comprehensively predict potential risks of the treatment.

## Data Availability

The original contributions presented in the study are included in the article/supplementary material, further inquiries can be directed to the corresponding authors.
